# Editorial: Lymphocyte Functional Crosstalk and Regulation

**DOI:** 10.3389/fimmu.2019.02916

**Published:** 2019-12-10

**Authors:** Raghvendra M. Srivastava, Francesco M. Marincola, Anil Shanker

**Affiliations:** ^1^Immunogenomics and Precision Oncology Platform, Memorial Sloan Kettering Cancer Center, New York, NY, United States; ^2^Refuge Biotechnologies Inc., Menlo Park, CA, United States; ^3^Department of Biochemistry, Cancer Biology, Neuroscience and Pharmacology, School of Medicine, Meharry Medical College, Nashville, TN, United States; ^4^Host-Tumor Interactions Research Program, Vanderbilt-Ingram Comprehensive Cancer Center, Vanderbilt University School of Medicine, Nashville, TN, United States; ^5^Vanderbilt Center for Immunobiology, Vanderbilt University School of Medicine, Nashville, TN, United States; ^6^Vanderbilt Institute for Infection, Immunology and Inflammation, Vanderbilt University School of Medicine, Nashville, TN, United States

**Keywords:** immune crosstalk, lymphocytes, T-cells, NK cells, dendritic cells, immunotherapy, cancer, infections

Lymphocyte effector responses constitute a key function of the vertebrate immunological defense. The responses are tightly controlled by a range of intracellular and intercellular mechanisms. A vast body of research demonstrates that the full potential of lymphocyte effector function is acquired following communications between the components of innate and adaptive immunity organized into an architecture of an interactive social network (1). For example, besides classical T-DC interactions (2–5), an interplay between dendritic cells (DC) and NK cells (6, 7), or T-cells and NK cells (8–18), plays important roles in resolving infections, solid cancers, or other pathologies. NK-T-regulatory (Treg) cell interplay maintains liver homeostasis (18). Furthermore, a tripartite crosstalk between T lymphocytes, DC, and NK cells is important for effector immune responses (19, 20). NK activity is also linked to T-cell and humoral responses (21). A cooperation between CD4^+^ and CD8^+^ T-cells during the effector phase has been suggested to eliminate bystander cancer cells (22). Thus, it is imperative to study immune compartments as an interdependent functional unit (23). A systematic understanding of the interaction between innate immune cells, innate lymphoid cells, including NK, and adaptive immune cells, such as T and B-cells can lead to improved immunotherapy approaches.

In pathologies of cancer, autoimmunity, chronic infections with prolonged inflammation, or tissue damage, lymphocytes are either compromised or they respond overtly. Immune dysfunction is linked to skewed immune cell distribution across several subtypes, including CD8^+^T, CD4^+^T, Treg, NK, innate lymphoid cells, DC, macrophages, neutrophils, or myeloid-derived suppressor cells (MDSC). Moreover, the efficiency of their crosstalk, and the frequency of intermediary players have a considerable role in determining the disease severity. Single cell and next generation sequencing technologies are revealing that numerous immune subtypes including previously uncharacterized subsets are affected in these diseases. Further, layers of underlying immune resistance and escape mechanisms interfere with the clinical outcomes, indicating our poor understanding of disease microenvironments and immune cell networkings.

This Research Topic was developed to gain an overview of these timely issues in lymphocyte biology with a particular emphasis on functional crosstalk and its regulation. In this topic, a series of articles ranging from basic science to translational and clinical reports, including a few very insightful reviews, provides meaningful insights toward this interesting field. These articles strongly support the premise that harnessing the immune cell crosstalk in immune disorders and cancer may uncover novel strategies to cure these diseases more effectively.

To improve the outcome of T-cell immunotherapy in cancer patients, immune checkpoint inhibitors targeting PD-1/PD-L1, or CTLA-4 pathways received a lot of excitement in the past decade. Checkpoint antibodies were developed and approved by the Food and Drug Administration (FDA) in the USA and Europe. A blockade of T-cell exhaustion or contraction molecules, PD-1/PD-L1 and CTLA-4, respectively, should reactivate and expand cancer antigen-specific T-cells. However, clinical responses were heterogeneous to none, with several resistance mechanisms identified across cancer types. Surprisingly, a hyperprogression was also reported recently in multiple cancer types, including melanoma and non-small cell lung cancer, possibly mediated by antibody-Fc/FcR interaction and amplification of PD-1^+^ Treg-cells (24–27). Seliger reviewed crucial factors pertaining to the limited efficacy of cancer immunotherapy, such as crosstalk between immune cells and gut microbiome, tumor-infiltrating regulatory myeloid cells, and the role of several immune cell subsets. The author also discussed how tumor mutational burden and neoantigen load regulates clinical outcomes (28). Limited success of negative immune regulators, i.e., PD-1 and CTLA-4, initiated the hunt for other promising negative checkpoint receptors and ligands. Several B7 molecules also regulate anti-tumor immunity (29). Included here, Cui et al. reported a novel B7-related molecule CD300c expressed on antigen-presenting cells, including B-cells, monocytes, macrophages, and DC. Its putative counter receptors were also identified on resting and activated CD8^+^ and CD4^+^ T-cells. They showed that CD300c blockade may reinvigorate T-cell responses. B- and T-lymphocyte attenuator (BTLA) is another crucial immunoregulatory receptor. Yu et al. discussed BTLA and its ligand HVEM (herpes virus entry mediator) signaling pathways, and highlighted chronological research needed in BTLA field. The widespread expression of BTLA across T, B, NKT, and DC indicates potential role of BTLA in immune crosstalk that could be harnessed to balance lymphocyte function and counteract immune disorders. The intracellular second messenger cyclic-AMP (cAMP) that acts in immunosuppressive signaling in T-cells is upregulated by multiple tumor-derived suppressive factors, such as prostaglandin E2 and adenosine. Schmetterer et al. show that ectopic overexpression of phosphodiesterase 4A (PDE4A) in T-cells leads to efficient degradation of cAMP. They suggest that PDE4A can be exploited as an immune checkpoint inhibitor against multiple suppressive factors.

Recently, the FDA approved chimeric antigen receptor (CAR) T-cell therapy for relapse and refractory B-cell acute lymphoblastic leukemia and diffuse large B-cell lymphoma following their success in multiple phase-I/II clinical trials. While CAR T-cells are considered as major breakthrough in the field of cancer immunotherapy, the regulation of CAR T-cells remains poorly understood. Dwivedi et al. reviewed the strategies that regulate CAR T-cell efficacy and persistence with focus on roles of different structural component of CAR construct.

Tumor microenvironment exhibits hypoxic and non-hypoxic areas heterogeneously. Hypoxic area is immune-deserted and appears to be resistant to immune cell attacks. Hypoxia is often associated with high amounts of adenosine, which is generated following ATP degradation. Adenosine restrains the effector function of T and NK cells. Several reports have indicated that interference with the adenosine signaling may improve anti-tumor immunity, especially in conjunction with immune checkpoint inhibitors. In a comprehensive review directed toward targeting adenosine in cancer immunotherapy, Vigano et al. presented an illustrious overview of several pre-clinical and clinical strategies to inhibit the negative effects of adenosine for superior cancer control. They described several mechanisms to intersect adenosine effects impacting T-cells, DC, macrophages, NK, neutrophils, MDSC, stromal cells, and tumor cells. Further enhancing the cross-talk among various immune cells in a hostile tumor microenvironment, Karan introduced the emerging concept of targeting the inflammasome to improve antitumor immunity. Since a multitude of cytokines play a pivotal role in the regulation of immune cell function, inflammasome targeting would likely modulate the profile of inflammatory cytokines reducing immunosuppression at tumor sites. It would be interesting to see how ongoing studies on inflammasomes would benefit the field of cancer immunotherapy.

Several groups have detected B-cell infiltration into solid cancers. Kotlan et al. investigated tumor-infiltrating B-cells. They performed antibody repertoire analysis at the genetic level and identified disialylated glycosphingolipid as tumor antigens in melanoma. They generated a novel tool, single-chain variable (ScFV) antibody, and performed several other tests to confirm that tumor-infiltrated B-cells can recognize tumor-associated glycosphingolipid on melanoma and other solid cancers. Since tumor-derived ganglioside can suppress B-cell antibody production, this B cell-cancer cell crosstalk could be utilized as a therapeutic target.

NK cells and T-cells recognize tumor cells with distinct types of receptors; however, they utilize common effector molecules, such as TNFα, perforin, and granzymes to exert their cytotoxic action. Uzhachenko and Shanker presented an emerging perspective that bidirectional crosstalk, and membranous reorganization between these CTL and NK cells may enable them to effectively eradicate cancer and infections. From human immune network proteome database, it is apparent that NK and CTL can profusely interact with DC to prime and optimize their effector function. Moreover, DC, being the professional antigen-presenting cells, have been tested as antigen delivery vehicle in several clinical trials. Vujanovic et al. interrogated the immunological effects of autologous DC transduced with MART-1, tyrosinase, and MAGE-A6 melanoma tumor antigens in a phase-I clinical trial. Interestingly, they identified that systemic high dose IFN-α2β after DC vaccination modulates a unique CD56^dim^CD16^negative^ non-cytolytic NK subset in melanoma patients. This dominant immunoregulatory NK subset in tumor microenvironment appears to contribute to better clinical outcomes.

Exosomes mediate several immunoregulatory mechanisms. In an *in vitro* CTL activation model, Wu et al. report that CTL-derived exosomes can increase the effector function and proliferation of CTL activated by low affinity peptides. The exosomes appear to mediate crosstalk among high and low-affinity CTLs at the beginning of CTL response. This may increase the repertoire of low affinity CTL and afford their long-term sustenance, thereby significantly improving T-cell mediated anti-cancer responses. In a mouse model, Kim et al. demonstrated that IL-7 and IL-15 cytokine-based crosstalk regulates the proportion and survival of naïve and memory T-cell populations. In the context of spontaneous proliferation of T-cells, Kim et al. showed an antigen-independent but IL-2-dependent crosstalk between CD4^+^ and CD8^+^T-cells.

T-cells also play a central role in modulating adipose tissue inflammation. In people living with HIV infection, Wanjalla et al. correlated subcutaneous adipose tissue with significantly increased frequency of CD4^+^ and CD8^+^ T-effector-memory and effector-memory-RA^+^ cells. Adipose tissue from HIV-infected individuals showed a higher expression of TLR2, TLR8, and multiple chemokines relevant to immune cell homing compared to HIV-negative controls with similar glucose tolerance.

The metabolic reprogramming of T cells by immunosuppressive drugs controls several inflammatory disorders (30). In humans infected with cytomegalovirus (CMV), Bak et al. showed significantly improved CMV-specific effector-memory T-cell function following inhibition of mammalian target of rapamycin (mTOR) with sirolimus. Monitoring of TCR-repertoire dynamics by next generation sequencing confirmed that the increased functionality was not related to sirolimus-resistant CTL-clones. Instead, environmental cues during CMV-CTL development *via* IL-2 receptor-driven signal transducer and activator of transcription-5 (STAT-5) signaling under mTOR inhibition allowed fine-tuning of T-cell programming for enhanced antiviral responses with stable TCR-repertoire dynamics. In a non-interventional prospective clinical trial in patients with multiple trauma, Hefele et al. assessed the role of platelets, Treg and Th17 cells in the post-traumatic immune response. They observed increased IL-17A expression in Th17 and Treg during the first 10 days following trauma. Moreover, despite a rising number of platelets, their analysis showed post-traumatic platelet dysfunction. Further studies are necessary to understand the underlying functional crosstalk between T-cells and platelets.

Recently, IL-9-producing Th9 cells have been identified in several disorders including cancer. Roy and Awasthi presented evidence that extracellular ATP promotes the differentiation of Th9 cells. They proposed a “*feed-forward loop model”* where nitric oxide production in Th9 cells enhances the mTOR-HIF-1-α pathway that further culminates in Th9 cell differentiation. This finding may have major implications in several cancer types. The role of Th9 cells in cancer is currently under investigation in several studies. Gaudino and Kumar described the crosstalk of T-cells and APC at multiple layers and presented a schematic of dysbiosis and gut microbiome. They highlighted how intestinal IL-17 receptor signaling and reciprocal crosstalk with gut microbiota can regulate autoimmunity. A comprehensive understanding of molecular interactions of immune cells and cytokines is important to refine appropriate immunotherapies. Dayakar et al. summarized the current understanding of a wide spectrum of cytokines and their interaction with immune cells that determine the clinical outcome of visceral leishmaniasis. They also highlighted opportunities for the development of novel diagnostics and intervention therapies for leishmaniasis.

Upadhyay describes the novel role of Ly6 gene family in cancer immune regulation. Ly6 genes are scattered in various chromosomes in human genome and have similarity to stem cell antigen-1 gene, a well-known cancer stem cell marker. The overexpression of this class of genes in solid cancers leads to poor survival. Some of these genes are expressed on both cancer cells and innate immune cells. Further research is necessary to understand if the Ly6 family genes could play a role in innate and adaptive immune crosstalk in the context of solid tumor microenvironments. Macrophage polarization is involved in many pathologies such as anti-cancer immunity and autoimmune diseases. Polarized macrophages exhibit plasticity when M2 macrophages are reprogrammed into an M1-like phenotype following treatment with IFNγ and/or LPS. At the same time, M1 macrophages are resistant to reprogramming in the presence of M2-like stimuli (IL-4). Veremeyko et al. explored the role of early growth response (Egr) family of transcriptional regulators in the induction and maintenance of M1 and M2 polarization. They demonstrated that a molecular crosstalk between Egr2 and CEBPβ transcription factors regulated macrophage polarization under distinct inflammatory conditions. An original study by Khare et al. investigated a switch of proximal and distal promoters fine-tuning the expression of genome organizer, special AT-rich sequence-binding protein (SATB1), which plays a crucial role in expression of multiple genes in a cell type-specific manner during the thymic development and peripheral differentiation and polarization of T-helper cells. Cytokine and TCR signaling crosstalk impacts SATB1 alternative promoter usage.

Collectively, the articles contained within the Research Topic highlight the leading concept of lymphocyte functional crosstalk and its underlying complexity that impacts disease pathology and outcomes. Further research into the functional dynamics of immune networks is essential and timely for advancing our understanding of the immunological basis of diseases and the design of preventive or therapeutic approaches. The knowledge acquired from the published articles will contribute to meaningful insights for the development of more refined and novel immune strategies.

## Author Contributions

RS and AS conceived, designed, and wrote the manuscript. FM provided substantial intellectual feedback. All authors read and approved the final manuscript for publication.

### Conflict of Interest

FM is employed by the company Refuge Biotechnologies Inc., Menlo Park, CA. FM is also on the Advisory Boards of Calidi Biotherapeutics Inc., San Diego, CA and CHIPSA^TM^ Hospital, Playas Tijuana, Baja California, Mexico. The remaining authors declare that the research was conducted in the absence of any commercial or financial relationships that could be construed as a potential conflict of interest.
